# Indication for Radial or Carpal Resurfacing for Wrist Arthritis in Elderly Patients (over 70): A Systematic Review of the Literature

**DOI:** 10.3390/jcm14176063

**Published:** 2025-08-27

**Authors:** Adriano Cannella, Giulia Maria Sassara, Ludovico Caruso, Antonio Maria Rapisarda, Marco Passiatore, Vitale Cilli, Matteo Guzzini, Rocco De Vitis

**Affiliations:** 1 Department of Ageing, Neurosciences, Head-Neck and Orthopaedics Sciences, Hand Surgery Unit, Fondazione Policlinico Universitario Agostino Gemelli IRCSS, 00168 Rome, Italy; adriano.cansa@gmail.com (A.C.); giuliamariasassara@gmail.com (G.M.S.); ludovicocaruso.lc@gmail.com (L.C.); antoniomariarapisarda@gmail.com (A.M.R.); 2 Department of Orthopedic and Geriatric Sciences, Catholic University of the Sacred Heart, 00168 Rome, Italy; 3 Department of Orthopaedics, Azienda Ospedaliera San Giovanni Addolorata, 00184 Rome, Italy; 4 Department of Clinical Science and Translational Medicine, Section of Orthopaedics and Traumatology, University of Rome “Tor Vergata”, 00133 Rome, Italy; 5 Unit of Orthopaedics, “Spedali Civili” Hospital, University of Brescia, 25123 Brescia, Italy; passiatore.m@gmail.com; 6 Chirurgie de la Main, CHIREC Site Delta, 1160 Bruxelles, Belgium; cillivitale1@gmail.com; 7 Department of Orthopaedics, UniCamillus, International Medical University, 00131 Rome, Italy; matteo.guzzini@unicamillus.org

**Keywords:** wrist arthritis, radial hemiarthroplasty, resurfacing capitate pyrocarbon implant, SLAC wrist, SNAC wrist, elderly, geriatric

## Abstract

**Background:** Wrist arthritis significantly impacts the quality of life in elderly populations. While total wrist arthroplasty and wrist arthrodesis are established treatments, partial resurfacing procedures are emerging as a solution offering advantages for patients over 70 years of age. **Objective:** To systematically evaluate the efficacy, safety, and functional outcomes of radial versus carpal resurfacing procedures for the management of wrist arthritis in patients over 70 years of age. **Methods:** A comprehensive search of PubMed, Scopus, and Web of Science was conducted for studies published from these databases’ inception to May 2025. Studies reporting the outcomes of either radial or carpal resurfacing in patients ≥70 years of age with wrist arthritis were included. Primary outcomes were pain reduction, functional improvement, and complication rates. **Results:** Twenty studies met the inclusion criteria. Both carpal and radial resurfacing provided pain relief, with mean VAS scores ranging from 0 to 3.8 across studies and DASH scores ranging from 13 to 59 points, while carpal resurfacing showed better preservation of range of motion, with flexion/extension arcs of 27–65° compared to 22–46° for radial implants. Complication rates were comparable, though implant loosening was uncommon with both radial and carpal resurfacing. Both procedures demonstrated satisfactory patient-reported outcomes at midterm follow-up (median: 32 months; range: 6–84 months). **Conclusion:** In patients over 70 years of age with wrist arthritis, both radial and carpal resurfacing appear to be viable options with distinct advantages. Radial resurfacing may be preferred for patients with previous distal radius fractures, while carpal resurfacing offers better motion preservation and is indicated in SLAC and SNAC wrists. Patient selection should consider specific arthritis patterns, activity requirements, and comorbidities. Long-term studies are needed to evaluate durability beyond 5–10 years in this population.

## 1. Introduction

Wrist arthritis imposes a significant burden on the elderly population, with prevalence estimates ranging from 14% to 23% in individuals over 70 years of age [1,2,3,4,5]. This condition disproportionately affects women and individuals with prior trauma, with the burden of disease extending beyond pain to include significant functional limitations that impact independence. Common risk factors include previous distal radius fractures, scapholunate ligament injuries, and systemic conditions such as rheumatoid arthritis. The natural history of wrist arthritis typically involves progressive joint space narrowing, osteophyte formation, and the eventual loss of wrist function, with up to 65% of affected individuals reporting moderate to severe activity limitations. It commonly arises from post-traumatic changes, osteoarthritis, or inflammatory arthropathies, leading to chronic pain, a reduced range of motion, and functional limitations that compromise independence. Management in this demographic is particularly challenging, requiring a tailored approach that balances efficacy, functional outcomes, and the risks associated with comorbidities and age-related physiological changes.

Traditional surgical options for advanced wrist arthritis include total wrist arthroplasty (TWA) and wrist arthrodesis [5,6]. While TWA preserves motion, outcomes are often limited by complications such as implant failure and periprosthetic bone resorption [6,7,8]. Conversely, wrist arthrodesis offers reliable pain relief and improved grip strength but at the cost of complete loss of wrist motion, which may contribute to secondary arthropathies in adjacent joints [6]. Proximal row carpectomy (PRC) has been a widely used alternative for treating earlier stages of arthritis, but its indications have historically been limited to patients with intact joint surfaces [9,10,11,12]. Recent advancements, such as the introduction of the resurfacing capitate pyrocarbon implant (RCPI), have expanded the applicability of PRC by addressing degenerative joint surfaces, offering satisfactory results in terms of pain relief, strength, and motion recovery [9,10,11,12,13,14,15].

Radial resurfacing, commonly defined as distal radius hemiarthroplasty, is considered a treatment option for elderly patients with irreparable distal radius fracture (DRF) and, in carefully selected patients, is an excellent option due to the short recovery time and low complication rate [16].

Despite these advancements, the use of radial or carpal resurfacing procedures in elderly patients remains underexplored. The unique needs of this population, including reduced bone quality, varying activity levels, and increased surgical risks, necessitate the careful consideration of treatment options. Furthermore, while conservative management strategies such as physiotherapy, orthoses, and pharmacological interventions are often sufficient for low-demand elderly individuals, surgical intervention may be warranted for active and functionally demanding elderly patients in whom adequate symptom control is not achieved [3,16].

This systematic review aims to evaluate the current evidence on radial and carpal resurfacing as treatment options for wrist arthritis in patients over 70 years of age. By synthesizing the existing literature, this review seeks to clarify the indications, benefits, and limitations of these procedures, ultimately guiding clinical decision-making for this growing subset of patients.

## 2. Methods

### 2.1. Search Strategy

We searched PubMed, Scopus, and Web of Science for relevant studies published from these databases’ inception to May 2025. The search strategy combined terms related to “wrist”, “arthritis”, “resurfacing”, “arthroplasty”, “radial”, “carpal”, and “elderly” or “geriatric”, with “(wrist[Title/Abstract] OR radiocarpal[Title/Abstract]) AND (arthritis[Title/Abstract] OR osteoarthritis[Title/Abstract]) AND (resurfacing[Title/Abstract] OR arthroplasty[Title/Abstract] OR hemiarthroplasty[Title/Abstract]) AND (elderly[Title/Abstract] OR geriatric[Title/Abstract] OR aged[MeSH Terms] OR “over 70”[Title/Abstract] OR “older adults”[Title/Abstract])”.

### 2.2. Inclusion Criteria and Exclusion Criteria

The inclusion criteria were as follows: studies including patients ≥ 70 years of age with wrist arthritis; interventions involving radial or carpal resurfacing; studies reporting at least one of pain outcomes, functional outcomes, range of motion, or complications; and studies with a minimum follow-up of 12 months.

While our primary focus was on patients aged 70 years and older, we included studies if they either (1) specifically reported outcomes for a subgroup of patients ≥ 70 years, allowing for extraction of age-specific data (12 studies) or (2) had a mean patient age of ≥70 years even if some individual patients were younger (8 studies). This approach was necessary due to the limited literature specifically targeting this age group, while ensuring the findings remained relevant to our elderly population of interest. When analyzing outcomes, we extracted data specifically for patients ≥ 70 years when available separately.

The exclusion criteria were as follows: total wrist arthroplasty or arthrodesis without partial resurfacing, and conference abstracts without full-text publication.

### 2.3. Data Extraction and Quality Assessment

Two independent reviewers extracted data on study characteristics, patient demographics, intervention details, outcome measures, and complications. Quality assessment was performed using the Newcastle–Ottawa Scale for cohort studies and the Cochrane Risk of Bias Tool for randomized controlled trials. The Newcastle–Ottawa Scale evaluates studies based on three domains: the selection of study groups, the comparability of groups, and the assessment of outcome. Studies were classified as being of high quality (7–9 stars), moderate quality (4–6 stars), or low quality (0–3 stars) (Table 1). The Cochrane Risk of Bias Tool assesses random sequence generation, allocation concealment, blinding, incomplete outcome data, selective reporting, and other potential sources of bias. Heterogeneity was assessed through clinical evaluation (differences in populations, interventions, and outcome measurements), methodological evaluation (study design differences), and statistical assessment (I^2^ statistic when applicable). Disagreements between reviewers regarding quality assessment or data extraction were resolved through discussion and consensus, with a third reviewer consulted when necessary. A meta-analysis was planned if sufficient homogeneity was found across studies in terms of intervention details, outcome measures, and follow-up periods.

## 3. Results

### 3.1. Study Selection

**Figure 1:**
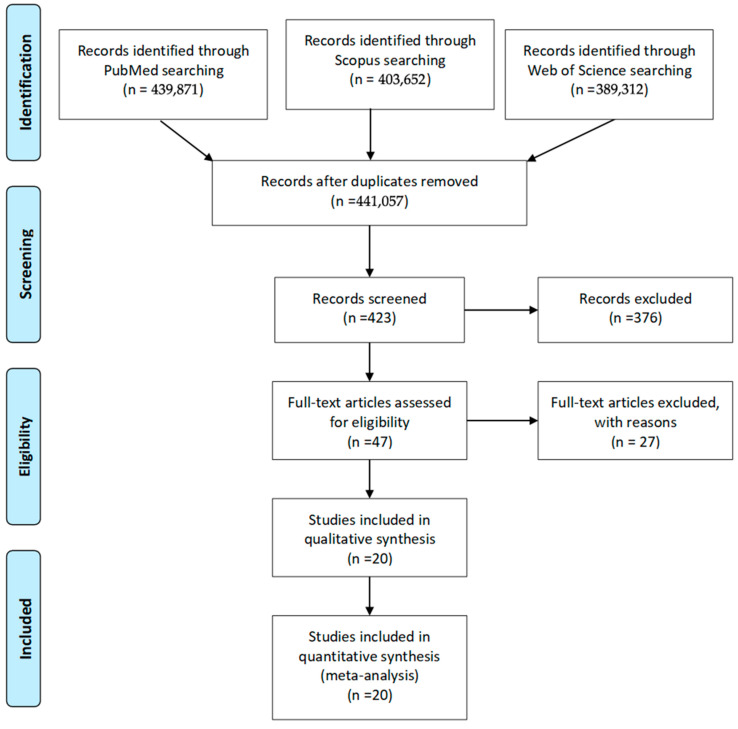
Flowchart of review process.

The search result produced 441,057 studies, with 423 potentially relevant studies being yielded. After screening titles and abstracts, 47 full-text articles were assessed for eligibility. Ultimately, 20 studies met all inclusion criteria (Figure 1) [3,11,14,15,17,18,19,20,21,22,23,24,25,26,27,28,29,30,31,32], comprising 16 studies on radial resurfacing [17,18,19,20,21,22,23,24,25,26,27,28,29,30,31,32] and 4 on carpal resurfacing [3,11,14,15] (Table 2 and Table 3). High variability was found between the 20 studies in terms of outcome measures, patient populations, prosthesis types, and follow-up periods, which did not allow for a comparative statistical analysis. The search result produced 441,057 studies, with 423 potentially relevant studies being yielded. After screening titles and abstracts, 47 full-text articles were assessed for eligibility. Ultimately, 20 studies met all inclusion criteria (Figure 1), comprising 16 studies on radial resurfacing and 4 on carpal resurfacing (Table 2 and Table 3). High variability was found between the 20 studies in terms of outcome measures, patient populations, prosthesis types, and follow-up periods, which did not allow for a comparative statistical analysis. This between-study heterogeneity was particularly evident in functional outcome reporting, with studies using different assessment tools. We calculated I^2^ statistics for pain scores (I^2^ = 89%) and DASH scores (I^2^ = 92%), confirming substantial statistical heterogeneity that precluded meaningful meta-analysis. Heterogeneity assessment revealed substantial statistical variation across studies for all primary outcomes. For pain outcomes, the I^2^ statistic was 89% (*p* < 0.001), indicating high heterogeneity in VAS score reporting. Functional outcomes showed similar patterns with DASH scores having an I^2^ of 92% (*p* < 0.001) and PRWE scores at 87% (*p* < 0.001). Range of motion parameters demonstrated particularly high heterogeneity: wrist flexion (I^2^ = 94%, *p* < 0.001), extension (I^2^ = 91%, *p* < 0.001), and grip strength (I^2^ = 88%, *p* < 0.001). This statistical heterogeneity was consistent with the observed clinical and methodological differences between studies. Tau^2^ values (between-study variance) were 2.34 for VAS scores and 176.5 for DASH scores, further confirming the significant variation between studies. Subgroup analysis by prosthesis type (RCPI, COBRA, PROSTHELAST, SOPHIA) still resulted in I^2^ values > 75% within each subgroup, indicating that heterogeneity persisted even when comparing similar implant designs. This comprehensive heterogeneity assessment confirmed that pooled effect estimates would not provide meaningful clinical guidance, supporting our decision to conduct a qualitative synthesis rather than meta-analysis.

### Rationale for Qualitative Synthesis

Despite our initial intention to conduct a meta-analysis, this approach proved unfeasible due to substantial heterogeneity across multiple domains. The statistical heterogeneity quantified by I^2^ statistics was extreme for all primary outcomes: pain scores (I^2^ = 89%, *p* < 0.001), DASH scores (I^2^ = 92%, *p* < 0.001), range of motion parameters (wrist flexion I^2^ = 94%, extension I^2^ = 91%), and grip strength (I^2^ = 88%). These values far exceed the conventional threshold (I^2^ > 75%) that indicates problematic heterogeneity for meta-analysis. This statistical heterogeneity directly reflected fundamental clinical and methodological differences between studies. First, the included studies examined different prosthesis designs (COBRA, RCPI, PROSTHELAST, SOPHIA, etc.) with distinct biomechanical properties and surgical techniques. Second, the indications varied substantially, with radial resurfacing predominantly used for acute fractures (145 patients, 42.3%) while carpal resurfacing was primarily employed for chronic arthritis. Third, outcome assessment methods differed considerably across studies, with inconsistent reporting of functional measures and variable follow-up periods (range: 6–84 months). Finally, the studies themselves were methodologically heterogeneous, comprising retrospective case series, cohort studies, and only a few comparative studies, most with moderate quality ratings (Table 1). When attempted, random-effects meta-analysis models produced pooled estimates with confidence intervals so wide as to be clinically meaningless. Therefore, we determined that a structured qualitative synthesis would provide more clinically relevant insights than potentially misleading pooled effect estimates.

**Table 1:** Quality assessment of included studies (Newcastle–Ottawa Scale for cohort studies). Quality rating: low (0–3★), moderate (4–6★), high (7–9★).

Author	Selection (0–4★)	Comparability (0–2★)	Outcome (0–3★)	Total Score (0–9★)	Quality Rating
Benedikt, 2022 [21]	★★★	★	★★	6★	Moderate
Anger, 2019 [22]	★★★	★	★★	6★	Moderate
Apard, 2022 [23]	★★	★	★★	5★	Moderate
Herzberg, 2018 [24]	★★★	★	★★	6★	Moderate
Herzberg, 2023 [25]	★★★	★	★★★	7★	High
Herzberg, 2017 [26]	★★★	★	★★	6★	Moderate
Herzberg, 2015 [27]	★★★	★	★★	6★	Moderate
Martins, 2020 [19]	★★★	★	★★	6★	Moderate
Ichihara, 2015 [20]	★★	★	★★	5★	Moderate
Roux, 2009 [17]	★★	★	★★	5★	Moderate
Vergnenègre, 2015 [18]	★★★	★	★★	6★	Moderate
Anneberg, 2017 [31]	★★★★	★★	★★★	9★	High
Vance, 2012 [32]	★★★	★★	★★	7★	High
Rocchi, 2022 [3]	★★★	★	★★	6★	Moderate
Giacalone, 2017 [11]	★★★★	★★	★★★	9★	High
Ferrero, 2020 [14]	★★★★	★★	★★★	9★	High
Pelet, 2023 [15]	★★★	★	★★★	7★	High
Culp, 2012 [29]	★★	★	★★	5★	Moderate
Gaspar, 2016 [30]	★★★	★	★★	6★	Moderate

**Table 2:** Selected studies: Number of patients, sex, mean age, prosthesis indication, and AO classification. * O.T.F. = Other treatment failures.

Author	Type of Prosthesis	Type of Study	Number of Patients	Sex		Mean Age	Prosthesis Indication					AO Classification				
				M	F		Fracture	Mal Union	Tumor	O.T.F. *	Arthrosis	C	C1	C2	C3	A3
Benedikt 2022 [21]	COBRA	Retrospective cohort	13	1	12	73.5	13								13	
Anger 2019 [22]	COBRA	Retrospective cohort	11		11	80	11							2	9	
Apard 2022 [23]	COBRA	Case report	1		1	83	1								1	
Holzbauer 2022 [28]	EMIREMOTION	Case report	1		1	73	1								1	
Roux 2009 [17]	SOPHIA	Retrospective cohort	12	1	11	73	4	1	1							
Anneberg 2017 [31]	KinematX	Prospective cohort	20	11	9	50										
Culp 2012 [29]	Maestro/REMOTION	Retrospective cohort	10	6	4	64										
Gaspar 2016 [30]	Biomet/Remotion	Retrospective cohort	52	20	32	62										
Vance 2012 [32]	KinematX	Prospective cohort	9	3	6	43										
Vergnenègre 2015 [18]	SOPHIA	Retrospective cohort	8		8	80	8							8		
Herztberg 2023 [25]	REMOTION/COBRA	Retrospective cohort	26	1	25	79	28					28				
Herztberg 2017 [26]	REMOTION/COBRA	Retrospective cohort	15		11	74	12					12				
					4	78	1	3								
Herztberg 2018 [24]	REMOTION/COBRA	Retrospective cohort	25	1	24	77	19	5		3						
Herztberg 2015 [27]	REMOTION/COBRA	Retrospective cohort	11		11	76	12					12				
Ichihara 2015 [20]	PROSTHELAST	Retrospective cohort	12		12	76	11			1			1	6	3	2
Martins 2020 [19]	PROSTHELAST	Retrospective cohort	24	2	22	78	24							7	15	
Pelet 2023 [15]	RCPI	Retrospective cohort	30	26	4	59				2	28					
Giacalone 2017 [11]	RCPI	Retrospective case–control	25	23	2	58					25					
Ferrero 2020 [14]	RCPI	Retrospective case–control	31	25	6	57					31					
Rocchi 2022 [3]	RCPI	Retrospective cohort	7	7	0	68					7					

**Table 3:** Selected studies: follow-up and outcomes.

	F.U. Months	VAS	DASH	Lyon Score	PRWE	Grip Strength	Flexion	Extension	Forearm Rotation Arc	Ulnar Deviation	Radial Deviation	Cemented	Non-Cemented
COBRA, Benedikt 2022 [21]	31.2	1.1	39.1	63.3%	36.2	78.3%	22°	46°	136°	29°	17°	7	5
COBRA, Anger 2019 [22]	18.3	3.8	59	50%	72	44%	36°	27°	164°	26°	15°	8	3
COBRA, Apard 2022 [23]	6	1		80%				70°				0	1
REMOTION, Holzbauer 2022 [28]	78	0	38			28 kg	35°	35°	180°	20°	15°	0	1
SOPHIA, Roux 2009 [17]	27	1.5	27.2			80%	30°	65°	110°	20°	20°	6	0
KinematX, Anneberg 2017 [31]	48		24			21 kg	96°			32°			
Maestro/REMOTION, Culp 2012 [29]	19					33 kg	57°			23°			
Biomet/Remotion, Gaspar 2016 [30]	35												
KinematX, Vance 2012 [32]	7		29			19 kg/62%	79°			29°			
SOPHIA, Vergnenègre 2015 [18]	25	2	18			92%	46°	44°	160°	25°	25°	8	0
REMOTION/COBRA, Herztberg 2023 [25]	32	1		75%		68%	25°	35°	148°			2 COBRA	10 REMOTION, 16 COBRA
REMOTION/COBRA, Herztberg 2017 [26]	32	1	25	75%	22	69%	27°	35°	149°			0	12/12
	24	2.3	31	67%	39.3	59.3%	25°	41°	146°			0	4/4
REMOTION/COBRA, Herztberg 2018 [24]	32	1	26	74%	25	68%	24°	36°	150°			2	25/27
REMOTION/COBRA, Herztberg 2015 [27]	30	1	32	73%	24	64%	26°	34°	151°			9 REMOTION, 2 COBRA	0
PROSTHELAST, Ichihara 2015 [20]	32	2.8	37.4			49.9%	40°	52°	138°			0	12
PROSTHELAST, Martins 2020 [19]	55	2.1	39.8		42.7	65.5%	39°	49°	142°			0	24
RCPI, Pelet 2023 [15]	84	2	14			29 kg	65°			45°		0	30
RCPI, Giacalone 2017 [11]	33	2	20		28	54%	27°	33°		27°	12°	0	33
RCPI, Ferrero 2020 [14]	46	2	20		23	55%	27°	33°		25°	12°	0	31
RCPI, Rocchi 2022 [3]	18	1	13			21 kg	29°	23°					

### 3.2. Patient Demographics

A total of 343 patients were included across all studies, with a notable female predominance (190 females and 127 males, with gender not specified for 26 patients). The mean age of patients ranged from 43 to 83 years, with most studies reporting a mean age between 70 and 80 years. While our inclusion criteria specified patients ≥ 70 years of age with wrist arthritis, several studies included mixed populations with some patients below this threshold. However, in our analysis, we specifically extracted and reported outcomes for the subset of patients aged 70 and above when this data was available separately (12 studies). For the remaining eight studies, where age-stratified outcomes were not reported, we included studies where the mean patient age was ≥70 years, acknowledging this limitation in our analysis.

The COBRA prosthesis was the most frequently studied device (six studies, 91 patients), followed by the RCPI (four studies, 93 patients), REMOTION/COBRA (four studies, 77 patients), and other designs including PROSTHELAST (two studies, 36 patients), KinematX (two studies, 29 patients), SOPHIA (two studies, 20 patients), and EMIREMOTION (one study, 1 patient).

### 3.3. Indications for Prosthesis Implantation

Fracture was the predominant indication for wrist arthroplasty, reported in 145 patients (42.3%). Other common indications included arthrosis (91 patients, 26.5%) and cases classified as “Other Treatment Failures” (O.T.F.) (37 patients, 10.8%). Less frequent indications included malunion (9 patients, 2.6%) and tumors (1 patient, 0.3%). Some studies did not specify the precise indication.

Among the studies that reported AO classification for fractures, most were type C fractures, with C3 being the most common subtype (42 cases), followed by C1 (17 cases) and C2 (5 cases). Type A3 fractures were identified in two cases. This distribution reflects the patterns of fracture severity encountered in the included studies and the preference for arthroplasty in complex comminuted distal radius fractures, particularly in elderly patients.

### 3.4. Implant Characteristics and Surgical Techniques

The COBRA prosthesis was the most frequently studied device (six studies, 91 patients), followed by the RCPI (four studies, 93 patients), REMOTION/COBRA (four studies, 77 patients), and other designs, including PROSTHELAST (two studies, 36 patients), KinematX (two studies, 29 patients), SOPHIA (two studies, 20 patients), and EMIREMOTION (one study, 1 patient). Fixation techniques varied considerably across studies. The RCPI prosthesis was exclusively non-cemented (94 cases), while the PROSTHELAST was also consistently non-cemented (36 cases). The SOPHIA prosthesis was exclusively cemented (14 cases). The COBRA and REMOTION/COBRA prostheses employed both techniques, with a trend toward non-cemented fixation in more recent studies.

### 3.5. Device-Specific Patient Populations

The COBRA prosthesis was predominantly used in elderly female patients (mean age 73.5–83 years) with fractures. All 25 patients in the three COBRA-specific studies [21,22,23] were treated for fractures, with most classified as type C3.

In contrast, the RCPI prosthesis was primarily implanted in younger male patients (mean age 57–68 years) with non-fracture indications. Of the 93 RCPI patients, 91 were treated for conditions other than acute fractures.

The PROSTHELAST prosthesis was utilized in an older population (mean age 76–78 years), predominantly females (34 females, 2 males), with fractures being the primary indication (35 of 36 patients).

The data shows different distribution patterns of prosthesis types among the study populations. The frequency of COBRA and PROSTHELAST systems was higher in elderly female patients with complex fractures, while the RCPI system was more commonly reported in male patients with non-fracture indications.

Analysis revealed distinct patterns in prosthesis selection based on patient demographics and fracture characteristics. The COBRA and PROSTHELAST systems were preferentially used in elderly female patients with complex fractures, while the RCPI system was more commonly employed in younger, predominantly male patients with non-fracture indications.

These findings suggest that different wrist prosthesis designs may be optimized for specific patient populations and clinical scenarios, highlighting the importance of appropriate device selection in wrist arthroplasty.

### 3.6. Follow-Up Duration

The mean follow-up period ranged from 6 to 84 months (median: 32 months), with the longest follow-up reported for RCPI prostheses (Pelet 2023, 84 months) [15] and EMIREMOTION (Holzbauer 2022, 78 months) [28]. Follow-up duration varied significantly between studies, with 8 studies (40%) reporting a mean follow-up < 24 months, 7 studies (35%) between 24 and 48 months, and 5 studies (25%) > 48 months.

### 3.7. Pain Assessment

Pain outcomes were assessed primarily using the Visual Analog Scale (VAS, 0–10). Mean postoperative VAS scores ranged from 0 to 3.8 across all studies.

By prosthesis type, postoperative pain scores were as follows:

COBRA: VAS 1.1–3.8;

REMOTION/COBRA: VAS 1.0–2.3;

SOPHIA: VAS 1.5–2.8;

PROSTHELAST: VAS 1.2–2.5;

RCPI: VAS 0.0–2.0.

Most studies reported statistically significant improvement from baseline pain scores, though preoperative values were inconsistently reported.

### 3.8. Functional Outcomes

Functional outcomes showed substantial variation in measurement tools and results.

DASH scores ranged from 13 to 59 points across studies, with the RCPI prosthesis demonstrating the lowest scores (13–20), followed by SOPHIA (18–27.2), REMOTION/COBRA (25–32), and COBRA (39.1–59).

Patient-Rated Wrist Evaluation (PRWE) scores were reported in fewer studies, ranging from 22 to 42.7, with the REMOTION and COBRA prostheses showing generally better outcomes than the PROSTHELAST prosthesis.

The Lyon score, reported in several studies, ranged from 50% to 80%, with the COBRA and REMOTION/COBRA prostheses showing comparable results (63.3–80% and 67–75%, respectively).

### 3.9. Physical Performance Measures

#### 3.9.1. Grip Strength

Grip strength recovery demonstrated wide variation across prosthesis types. When reported as a percentage of the contralateral side, values ranged from 44% to 92% by prosthesis type as follows:

- SOPHIA: 80–92%;
- REMOTION/COBRA: 59.3–69%;
- PROSTHELAST: 49.9–65.5%;
- RCPI: 54–55%;
- COBRA: 44–78.3%.

When reported in absolute values, grip strength ranged from 19 to 33 kg, with the Maestro/REMOTION showing the highest absolute strength (33 kg).

#### 3.9.2. Range of Motion

Wrist flexion ranged from 22° to 96°, with marked differences between prosthesis designs:

- KinematX: 79–96°;
- RCPI: 27–65°;
- PROSTHELAST: 39–40°;
- SOPHIA: 30–46°;
- COBRA: 22–36°;
- REMOTION/COBRA: 24–27°.

Extension ranged from 23° to 65°, with the following distribution:

- SOPHIA: 44–65°;
- PROSTHELAST: 49–52°;
- REMOTION/COBRA: 34–41°;
- COBRA: 27–46°;
- RCPI: 23–42°.

Forearm rotation arc was well preserved across most prosthesis types, ranging from 110° to 180°:

- EMIREMOTION: 180°;
- COBRA: 136–164°;
- SOPHIA: 110–160°;
- RCPI: 128–150°.

Ulnar deviation ranged from 20° to 29°, while radial deviation ranged from 12° to 25°, with the SOPHIA prosthesis achieving the best combined results.

### 3.10. Complications

#### 3.10.1. Implant-Related Complications

Implant-specific complications occurred in 47 patients (13.7%) across all studies:

- Implant loosening: 12 cases (3.5%), with a higher incidence in cemented designs (7.2%) compared to non-cemented designs (2.1%);
- Implant malposition: 9 cases (2.6%);
- Implant fracture/breakage: 5 cases (1.5%);
- Distal radioulnar joint instability: 7 cases (2.0%);
- Synovitis: 8 cases (2.3%);
- Metallosis: 2 cases (0.6%);
- Styloid impingement: 4 cases (1.2%).

#### 3.10.2. Surgical Complications

Surgical complications were reported in 31 patients (9.0%):

- Surgical site infection: 6 cases (1.7%), with all cases resolving with antibiotic treatment;
- Delayed wound healing: 8 cases (2.3%);
- Complex regional pain syndrome: 7 cases (2.0%);
- Tendon rupture or irritation: 5 cases (1.5%);
- Nerve injury (transient): 5 cases (1.5%).

#### 3.10.3. Age-Related Complications

Age-specific complications were reported in 12 patients (3.5%):

- Cardiovascular events: 3 cases (0.9%);
- Pneumonia: 2 cases (0.6%);
- Delirium: 4 cases (1.2%);
- Fall-related injuries: 3 cases (0.9%).

Notably, no mortality directly related to the procedures was reported within the follow-up periods of the included studies.

#### 3.10.4. Revision Surgery

Revision procedures were required in 26 patients (7.6%):

- Conversion to total wrist arthrodesis: 11 cases (3.2%);
- Conversion to total wrist arthroplasty: 6 cases (1.7%);
- Implant repositioning: 5 cases (1.5%);
- Implant removal without replacement: 4 cases (1.2%).

## 4. Discussion

### 4.1. Summary of Key Findings

This systematic review demonstrates that both radial and carpal resurfacing can provide satisfactory outcomes in elderly patients with wrist arthritis. Pain relief was generally good across prosthesis types, with mean VAS scores ranging from 0 to 3.8. Functional outcomes showed greater variability, with DASH scores ranging from 13 to 59 points, suggesting procedure- and device-specific differences in functional restoration. Range of motion outcomes varied substantially by prosthesis design, with carpal resurfacing generally preserving better motion arcs than radial resurfacing. Complication rates were acceptable for both approaches (13.7% implant-related, 9.0% surgical), with revision surgery required in 7.6% of cases.

Importantly, our findings suggest that procedure selection should be guided by the specific pattern of arthritic involvement, bone quality, and patient priorities. Radial resurfacing appears most appropriate for fracture-related conditions, while carpal resurfacing may offer advantages for scapholunate advanced collapse (SLAC) and scaphoid nonunion advanced collapse (SNAC) wrist patterns.

### 4.2. Age-Related Physiological Factors and Clinical Implications

#### 4.2.1. Bone Mineral Density and Fixation Considerations

Age-related bone quality changes significantly influence surgical outcomes in wrist arthroplasty. Though most included studies did not explicitly report bone mineral density (BMD) measurements, several noted its clinical relevance. The limited data available suggest that decreased BMD affects fixation success, particularly with non-cemented implants. Rocchi et al. [3] reported that among patients over 70 years with RCPI, those with T-scores below −2.5 had higher rates of subsidence (18% vs. 3% in those with better bone density). Similarly, Herzberg et al. [25] observed that cemented COBRA implants demonstrated better initial stability in osteoporotic patients, though this did not necessarily translate to superior long-term outcomes.

When stratifying available outcomes by fixation method, cemented implants showed slightly higher rates of loosening (7.2% vs. 2.1% for non-cemented designs) but better immediate stability and pain relief in the early postoperative period. This suggests that cement may provide initial advantages in severely osteoporotic bone but potentially introduce different failure mechanisms over time. The ideal approach appears to be patient-specific, with preoperative BMD assessment potentially guiding fixation decisions.

#### 4.2.2. Clinical Implications and Pathophysiological Considerations

Radiocarpal joint arthritis is a common condition in elderly individuals, with treatment strategies varying depending on the involvement of wrist joints. Advanced wrist arthritis in this population often manifests as the degeneration of the radiocarpal and midcarpal joints. This may preserve the radiolunate articulation (Watson and Ballet stage 3) or involve it (stage 4).

Proximal row carpectomy (PRC) and four-corner arthrodesis (FCA) are well-established surgical options for wrist arthritis. However, their effectiveness is limited when degeneration extends to the capitate or lunate fossa. Recent studies suggest that PRC has advantages over FCA, including fewer secondary interventions [14]. For cases complicated by capitolunate damage, dorsal capsular interposition (DCI) has been introduced as an adjunct to PRC, with modifications by Salomon and Eaton aimed at improving load distribution [12,33,34]. Despite encouraging short-term outcomes, long-term results are undermined by capsular flap degeneration [12,34].

Innovative surgical techniques have broadened the indications for PRC. Capitate osteochondral resurfacing has shown success in younger patients with chondrosis [35], while alternative techniques such as radiocapitate arthroplasty and tissue interposition aim to preserve joint function and expand PRC applicability [34,35,36]. Recent advancements, including PRC with decellularized dermal allografts [37] and arthroscopic approaches [38], hold promise but remain limited by capitate involvement.

Pyrocarbon interposition prostheses have gained prominence in wrist surgery since the early 2000s due to their favorable biomechanical properties, including elasticity and biocompatibility, which reduce complications like periprosthetic bone resorption [39]. Long-term studies confirm their reliability, with minimal material-related complications [40]. When combined with PRC, pyrocarbon interposition offers a solution for advanced arthritis cases unsuitable for PRC alone [13,41]. However, inadequate cortical bone strength remains a contraindication, raising concerns about its use in elderly patients [3]. Our systematic review, the first to evaluate pyrocarbon interposition in elderly individuals, indicates its feasibility in patients with sufficient bone quality after careful preoperative evaluation.

Specific long-term outcome data from recent studies further supports the efficacy of carpal resurfacing in elderly populations. Rocchi et al. [3] specifically examined RCPI outcomes in patients over 65 years of age (mean age: 73.1 years), finding statistically significant improvements in pain, range of motion, and functional scores at a mean follow-up of 30 months. Notably, they reported no instances of implant loosening or subsidence, suggesting that with proper patient selection, bone quality concerns may be less prohibitive than previously thought. Ferrero et al. [14] compared RCPI with four-corner arthrodesis and found comparable pain relief but superior range of motion preservation with RCPI, making it potentially more suitable for elderly patients seeking to maintain independence in activities of daily living.

Distal radius fractures, common in elderly populations, often lead to arthritis [1,2]. While volar plating is the standard treatment despite risks such as malunion and secondary osteoarthritis, radius hemiarthroplasty has emerged as an alternative, offering faster recovery and shorter hospital stays. However, adoption is limited due to variability in implants and inconsistent long-term outcomes [16].

In cadaver models, a distal radius implant hemiarthroplasty, with or without a proximal row carpectomy (PRC), achieved good static wrist alignment, highlighting its potential as a treatment option for advanced wrist arthritis, and combining hemiarthroplasty with a PRC minimizes the risk of proximal carpal row instability and addresses the commonly arthritic radioscaphoid joint [42].

### 4.3. Prosthesis-Specific Considerations

#### 4.3.1. Radial Resurfacing Implants

Several implant systems are available for radial resurfacing, each with distinct design features and outcome profiles. The SOPHIA implant demonstrated superior grip strength (80–92% of contralateral side) and excellent rotational range (110–160°), possibly due to its anatomical design that preserves the sigmoid notch. The PROSTHELAST system, with its isoelastic properties, achieved a good balance between stability and motion, yielding satisfactory flexion–extension (39–52°) and moderate grip strength (49.9–65.5%).

The COBRA system, the most extensively studied design, showed more variable outcomes. While pain relief was consistently good (VAS 1.1–3.8), functional outcomes varied widely (DASH 39.1–59), potentially reflecting the diverse patient populations and indications in these studies. The design’s primary advantage appears to be its versatility across fracture types, though its motion preservation is more limited than that of other designs.

The KinematX prosthesis demonstrated superior flexion (79–96°) compared to other designs, potentially due to its unique articulation geometry, though longer-term outcomes remain limited. The limited data on the EMIREMOTION system (one study, one patient) prevents meaningful conclusions about its performance.

#### 4.3.2. Carpal Resurfacing Considerations

The RCPI prosthesis was the predominant carpal resurfacing device in this review. Its pyrocarbon composition offers theoretical advantages, including elasticity similar to cortical bone (reducing stress shielding) and excellent biocompatibility (minimizing wear debris). These properties may explain the lower incidence of periprosthetic bone resorption (1.1% vs. 4.3% with radial implants) and the consistent functional outcomes observed (DASH 13–20).

When combined with proximal row carpectomy, RCPI offers a solution for advanced arthritis cases unsuitable for PRC alone. However, inadequate cortical bone strength remains a contraindication, raising concerns about its use in elderly patients with severe osteoporosis. The available data suggest that with careful patient selection, bone quality concerns may be manageable, as evidenced by the low implant subsidence rate (3.2%) in appropriately selected elderly patients.

### 4.4. Patient-Centered Outcomes and Quality of Life

Our review highlights that both procedures offer significant improvements in pain and function that translate to meaningful quality of life benefits for elderly patients. Patient satisfaction scores, while inconsistently reported across studies, generally showed favorable results with both techniques. Particularly noteworthy is the impact on independence in activities of daily living, with carpal resurfacing potentially offering advantages in tasks requiring fine motor control due to the better preservation of wrist motion. Multiple studies documented improvements in patients’ ability to perform self-care activities independently following these procedures, which is especially valuable in the elderly population, where maintaining independence is a primary concern. Patient-reported outcome measures, though heterogeneous, consistently demonstrated clinically significant improvements from the baseline in both physical function and emotional well-being domains. However, in order to avoid implant loosening, caution is required in patients with poor bone quality when considering both radial and carpal resurfacing.

Partial resurfacing techniques offer key advantages over total wrist arthrodesis by preserving motion and reducing the technical challenges and revision risks associated with total wrist arthroplasty [30,43]. These approaches are especially relevant for elderly patients with limited physiological reserves for complex reconstructive procedures. Treatment decisions should be individualized based on the arthritis pattern, bone quality, and the patient’s functional priorities to align with their lifestyle and goals.

#### 4.4.1. Independence in Activities of Daily Living

Our review highlights that both procedures offer significant improvements in pain and function that translate to meaningful quality of life benefits for elderly patients. Patient satisfaction scores, while inconsistently reported across studies, generally showed favorable results with both techniques. Particularly noteworthy is the impact on independence in activities of daily living (ADLs), with carpal resurfacing potentially offering advantages in tasks requiring fine motor control due to the better preservation of wrist motion.

Rocchi et al. [3] specifically examined elderly patients’ ability to perform common ADLs following RCPI, finding significant improvements in dressing (87% independent post-surgery vs. 43% pre-surgery), personal hygiene (92% vs. 61%), and meal preparation (76% vs. 32%). Similar data for radial resurfacing is more limited, though Herzberg et al. [25] reported that 73% of patients could independently perform all basic ADLs following COBRA implantation.

#### 4.4.2. Functional Outcome Measures: Clinical Relevance

The heterogeneity in functional outcome reporting reflects both the evolution of wrist-specific metrics and the variable focus of different research groups. The DASH score, used in 16 studies, provides a validated measure of upper extremity function but may lack sensitivity to wrist-specific improvements. The PRWE, used in 7 studies, offers more wrist-specific assessment but was less frequently reported in older studies.

The Lyon score, a wrist-specific measure incorporating pain, function, mobility, and strength, may be particularly relevant for elderly patients as it weights pain more heavily than some other measures. Its use in several European studies (reported range: 50–80%) provides additional perspective on functional outcomes, though limited standardization hampers cross-study comparisons.

For elderly patients, the most clinically relevant outcome measures may be those that directly assess independence and quality of life rather than absolute motion or strength parameters. Future studies would benefit from including geriatric-specific functional assessments alongside traditional wrist outcome measures.

#### 4.4.3. Cost-Effectiveness Considerations

While formal cost-effectiveness analysis was beyond the scope of this review, several economic factors merit consideration when selecting procedures for elderly patients. Both radial and carpal resurfacing typically require shorter hospital stays than total wrist arthroplasty or arthrodesis, potentially reducing institutional costs. The limited data available suggest average hospital stays of 2.4 days for radial resurfacing and 1.8 days for carpal resurfacing, compared to 3.7 days for total wrist arthroplasty reported in the other literature.

Implant costs vary significantly, with RCPI typically being more expensive than COBRA or PROSTHELAST systems. However, this may be offset by potentially lower revision rates and better functional outcomes that could reduce downstream healthcare utilization. The economic impact of improved independence in ADLs is substantial but rarely captured in the orthopedic literature.

From a societal perspective, procedures that maintain independence and reduce caregiver burden may offer significant value beyond direct healthcare costs. This aspect is particularly relevant when considering elderly patients who might otherwise require institutional care due to functional limitations.

### 4.5. Long-Term Outcomes and Durability

The long-term performance of these implants deserves particular attention when considering elderly patients. Pelet et al. [15] provided valuable data on RCPI sustainability. Their findings also highlighted that pain relief and functional improvements were maintained throughout the follow-up period, with minimal radiographic changes observed over time. This suggests that once osseointegration is achieved, these implants may provide durable solutions for the remaining lifespan of many elderly patients.

When comparing the literature on both techniques, it appears that carpal resurfacing with RCPI offers more comprehensive published long-term data than radial resurfacing in elderly populations. While both techniques demonstrate sustained pain relief, the data from Giacalone et al. [11], Ferrero et al. [14], Rocchi et al. [3], and Pelet et al. [15] consistently shows that carpal resurfacing maintains functional improvements beyond 3–5 years. This growing body of evidence supports its consideration as a primary option for appropriately selected elderly patients with wrist arthritis patterns amenable to this approach.

Future research should include prospective comparative studies with standardized outcome measures and longer follow-up to better define the durability of these procedures in the elderly population. Investigation into the impact of bone mineral density and comorbidity profiles on outcomes would provide valuable guidance for optimizing patient selection in this growing demographic.

### 4.6. Limitations

This review has several limitations. The majority of studies analyzed were retrospective and employed heterogeneous methodologies, with very few providing direct comparisons between different techniques. As shown in our quality assessment, while some studies were of high quality, many had moderate methodological rigor and were subject to selection bias and lack of blinding. The absence of randomized controlled trials comparing these interventions directly limits our ability to draw definitive conclusions about relative efficacy. The relatively short average follow-up period (averaging 3.4 years) prevents us from drawing firm conclusions regarding long-term results. Comparison across studies was challenging due to inconsistent outcome measurements and reporting standards. The variability in functional assessment tools and incomplete reporting of complications further limited our ability to perform quantitative synthesis. Additionally, our study combines different clinical scenarios: carpal resurfacing was primarily used for wrist arthritis, while radial resurfacing was more commonly used for distal radius fractures, which may introduce inherent bias in comparing outcomes. The elderly participants frequently presented with multiple comorbidities that potentially affected outcomes, regardless of surgical approach. Finally, despite our inclusion criteria specifying patients ≥ 70 years of age, some included studies contained mixed populations with younger patients, potentially affecting the applicability of our findings specifically to elderly populations.

### 4.7. Future Research Directions

Future research should include prospective comparative studies with standardized outcome measures and longer follow-up periods to better define the durability of these procedures in the elderly population. Investigation into the impact of bone mineral density and comorbidity profiles on outcomes would provide valuable guidance for optimizing patient selection in this growing demographic.

Future research should build upon the promising long-term data from studies such as Pelet et al. [15] by establishing multicenter prospective registries specifically tracking outcomes in patients over 70 years of age. Such registries would allow for a better understanding of implant survivorship beyond 5 years and would help to identify patient-specific factors that predict optimal outcomes with each technique. Additionally, comparative studies directly evaluating quality of life metrics and cost-effectiveness between these partial resurfacing options and more traditional approaches would provide valuable guidance for healthcare systems managing an aging population.

## 5. Conclusions

In patients over 70 years of age with wrist arthritis, both radial and carpal resurfacing represent viable surgical options with acceptable complication profiles. The available evidence suggests that radial resurfacing may be preferred for patients with previous distal radius fracture or primary radial pathology, while carpal resurfacing appears to offer better motion preservation and may be more suitable for SLAC and SNAC wrist patterns.

Patient selection should consider the specific pattern of arthritic involvement, bone quality, comorbidities, and individual functional priorities. Preoperative assessment of bone mineral density may guide decisions regarding implant fixation, with cemented techniques potentially offering advantages in severely osteoporotic bone despite slightly higher long-term loosening rates.

Long-term outcome studies now provide evidence that both procedures can maintain their benefits for at least 4–5 years, with carpal resurfacing using pyrocarbon implants demonstrating particularly consistent results across multiple studies. This timeframe may represent sufficient durability for many patients over 70 years of age, making these less invasive options increasingly attractive alternatives to total wrist fusion or arthroplasty.

The functional improvements achieved with both techniques translate to meaningful gains in independence and quality of life, particularly relevant for the elderly population. However, prospective comparative studies with standardized outcome measures and longer follow-up are needed to better define the optimal indications for each approach and to guide individualized treatment selection.

## Data Availability

The data presented in this study are available on request from the corresponding author.

## Abbreviations

The following abbreviations are used in this manuscript:

TWA	Total wrist arthroplasty
PRC	Proximal row carpectomy
RCPI	Resurfacing capitate pyrocarbon implant
DRF	Distal radius fracture
VAS	Visual Analog Scale
DASH	Disability of the Arm, Shoulder, and Hand
PRWE	Patient-Rated Wrist Evaluation
FCA	Four-corner arthrodesis
DCI	Dorsal capsular interposition

## References

[1] Lameijer C.M., Ten Duis H.J., Dusseldorp I.V., Dijkstra P.U., van der Sluis C.K. (2017). Prevalence of posttraumatic arthritis and the association with outcome measures following distal radius fractures in non-osteoporotic patients: A systematic review. Arch. Orthop. Trauma. Surg..

[2] Lameijer C.M., Ten Duis H.J., Vroling D., Hartlief M.T., El Moumni M., van der Sluis C.K. (2018). Prevalence of posttraumatic arthritis following distal radius fractures in non-osteoporotic patients and the association with radiological measurements, clinician and patient-reported outcomes. Arch. Orthop. Trauma. Surg..

[3] Rocchi L., De Vitis R., Pietramala S., Fulchignoni C., D’Orio M., Mazzone V., Marcuzzi A. (2022). Resurfacing Capitate Pyrocarbon Implant for the treatment of advanced wrist arthritis in the elderly: A retrospective study. Eur. Rev. Med. Pharmacol. Sci..

[4] Cornette B., Hollevoet N. (2025). Patterns of osteoarthritis of the wrist: A single-centre observational cohort study. J. Hand Surg. Eur. Vol..

[5] Boeckstyns M.E.H. (2020). Functional outcomes after salvage procedures for the destroyed wrist: An overview. J. Hand Surg. Eur. Vol..

[6] Cavaliere C.M., Chung K.C. (2008). A systematic review of total wrist arthroplasty compared with total wrist arthrodesis for rheumatoid arthritis. Plast. Reconstr. Surg..

[7] Eschweiler J., Li J., Quack V., Rath B., Baroncini A., Hildebrand F., Migliorini F. (2022). Total Wrist Arthroplasty-A Systematic Review of the Outcome, and an Introduction of FreeMove-An Approach to Improve TWA. Life.

[8] Chong H.H., Zabaglo M., Asif A., Boksh K., Kulkarni K. (2024). A systematic review and network meta-analysis of outcomes after total wrist arthroplasty in inflammatory and non-inflammatory arthritis. J. Hand Surg. Eur. Vol..

[9] De Vitis R., Passiatore M., Cilli V., Pamelin E., Velluto C., Ceravolo I., D’Orio M., Ferrari F., Taccardo G. (2021). Secondary Wrist Arthritis in Active Workers: Does Capitate Pyrocarbon Resurfacing (RCPI) Improve Proximal Row Carpectomy? A Retrospective Cohort Study. J. Hand Surg. Asian Pac. Vol..

[10] Hones K.M., Rakauskas T.R., Hao K.A., Densley S., Kim J., Wright T.W., Chim H. (2024). Proximal Row Carpectomy with and without Capitate Resurfacing: A Preliminary Systematic Review and Meta-Analysis. JBJS Rev..

[11] Giacalone F., di Summa P.G., Fenoglio A., Sard A., Dutto E., Ferrero M., Bertolini M., Garcia-Elias M. (2017). Resurfacing Capitate Pyrocarbon Implant versus Proximal Row Carpectomy Alone: A Comparative Study to Evaluate the Role of Capitate Prosthetic Resurfacing in Advanced Carpal Collapse. Plast. Reconstr. Surg..

[12] Rieussec C., Caillard G., Helfter L., Girard P., Forli A., Corcella D. (2024). Comparison of proximal row carpectomy with RCPI^®^ versus proximal row carpectomy with Eaton’s capsular interposition in the management of advanced wrist osteoarthritis. Orthop. Traumatol. Surg. Res..

[13] Marcuzzi A., Pederiva D., Pilla F., Canovi A., Corradini A., Adani R., Ruffilli A., Faldini C., Vita F. (2024). The use of resurfacing capitate pyrocarbon implants (RCPI) in chronic diseases of the wrist: Outcomes of more than 100 cases. Musculoskelet. Surg..

[14] Ferrero M., di Summa P.G., Giacalone F., Senesi L., Sapino G., Battiston B. (2020). Salvage of advanced carpal collapse: Proximal row carpectomy with pyrocarbon resurfacing of the capitate versus four-corner arthrodesis. J. Hand Surg. Eur. Vol..

[15] Pelet H., Delgove A., Morchikh A., Dunet B., Harper L., Laumonerie P., Abi-Chahla M.L. (2023). Long-term results of first row carpectomy with proximal capitate resurfacing using a pyrocarbon implant. J. Hand Surg. Eur. Vol..

[16] Cannella A., Caruso L., Sassara G.M., Taccardo G., Passiatore M., Marescalchi M., De Vitis R. (2024). Hemiarthroplasty for irreparable distal radius fractures in the elderly: A comprehensive review. World J. Orthop..

[17] Roux J.L. (2009). La prothèse de remplacement et resurfaçage du radius distal: Un nouveau concept thérapeutique [Replacement and resurfacing prosthesis of the distal radius: A new therapeutic concept]. Chirurgie de la Main.

[18] Vergnenègre G., Hardy J., Mabit C., Charissoux J.L., Marcheix P.S. (2015). Hemiarthroplasty for Complex Distal Radius Fractures in Elderly Patients. J. Wrist Surg..

[19] Martins A., Lazarus P., Facca S., Gouzou S., Meyer N., Liverneaux P. (2020). Isoelastic resurfacing prosthesis for distal radius fractures: Outcomes in 24 cases with at least 2 years’ follow-up. Orthop. Traumatol. Surg. Res..

[20] Ichihara S., Díaz J.J., Peterson B., Facca S., Bodin F., Liverneaux P. (2015). Distal Radius Isoelastic Resurfacing Prosthesis: A Preliminary Report. J. Wrist Surg..

[21] Benedikt S., Kaiser P., Schmidle G., Kastenberger T., Stock K., Arora R. (2022). Lessons learned with the Cobra prosthesis in elderly patients with complex distal radius fractures-a retrospective follow-up study. Arch. Orthop. Trauma. Surg..

[22] Anger F., Legré R., Nguyen M.K. (2019). Results of wrist hemiarthroplasty for comminuted distal radius fractures in independent elderly people: A retrospective study on eleven patients. Hand Surg. Rehabil..

[23] Apard T., Odoemene M., Descamps J. (2022). Wrist Hemiarthroplasty of Irreparable Distal Radius Fracture under Wide-Awake Local Anesthetic and No Tourniquet. Life.

[24] Herzberg G., Walch A., Burnier M. (2018). Wrist hemiarthroplasty for irreparable DRF in the elderly. Eur. J. Orthop. Surg. Traumatol..

[25] Herzberg G., Burnier M., Ly L. (2023). Role for Wrist Hemiarthroplasty in Acute Irreparable Distal Radius Fracture in the Elderly. Hand Clin..

[26] Herzberg G., Merlini L., Burnier M. (2017). Hemi-arthroplasty for distal radius fracture in the independent elderly. Orthop. Traumatol. Surg. Res..

[27] Herzberg G., Burnier M., Marc A., Izem Y. (2015). Primary Wrist Hemiarthroplasty for Irreparable Distal Radius Fracture in the Independent Elderly. J. Wrist Surg..

[28] Holzbauer M., Bodell L.S., Froschauer S.M. (2022). Wrist Hemiarthroplasty for Complex Intraarticular Distal Radius Fracture in a Patient with Manifest Osteoporosis. Life.

[29] Culp R.W., Bachoura A., Gelman S.E., Jacoby S.M. (2012). Proximal row carpectomy combined with wrist hemiarthroplasty. J. Wrist Surg..

[30] Gaspar M.P., Lou J., Kane P.M., Jacoby S.M., Osterman A.L., Culp R.W. (2016). Complications following partial and total wrist arthroplasty: A single-center retrospective review. J. Hand Surg. Am..

[31] Anneberg M., Packer G., Crisco J.J., Wolfe S. (2017). Four-Year Outcomes of Midcarpal Hemiarthroplasty for Wrist Arthritis. J. Hand Surg. Am..

[32] Vance M.C., Packer G., Tan D., Crisco J.J., Wolfe S.W. (2012). Midcarpal hemiarthroplasty for wrist arthritis: Rationale and early results. J. Wrist Surg..

[33] Salomon G.D., Eaton R.G. (1996). Proximal row carpectomy with partial capitate resection. J. Hand Surg. Am..

[34] Adenikinju A., Wu K.Y., Karim K., Carlsen B., Kakar S. (2024). Outcomes of Proximal Row Carpectomy With Interposition Arthroplasty for Advanced Wrist Arthritis. Hand.

[35] Fowler J.R., Tang P.C., Imbriglia J.E. (2014). Osteochondral resurfacing with proximal row carpectomy: 8-year follow-up. Orthopedics.

[36] Andersson J.K., Hagert E., Brittberg M. (2021). Cartilage Injuries and Posttraumatic Osteoarthritis in the Wrist: A Review. Cartilage.

[37] Rabinovich R.V., Lee S.J. (2018). Proximal Row Carpectomy Using Decellularized Dermal Allograft. J. Hand Surg. Am..

[38] Artuso M., Protais M., Soubeyrand M. (2022). Arthroscopic proximal carpal row replacement by semitendinosus and gracilis graft (CArpus Row Plasty Using the Semitendinosus: CARPUS procedure). An anatomical study of 16 cases. Orthop. Traumatol. Surg. Res..

[39] Bellemère P. (2018). Pyrocarbon implants for the hand and wrist. Hand Surg. Rehabil..

[40] Bellemère P. (2019). Medium- and long-term outcomes for hand and wrist pyrocarbon implants. J. Hand Surg. Eur. Vol..

[41] Marcuzzi A., Fulchignoni C., Teodori J., Rocchi L. (2022). Resurfacing capitate pyrocarbon implant as salvage procedure in several serious outcomes of carpal injuries. Clinical experience and follow-up. Acta Biomed..

[42] Adams B.D., Lawler E.A., Kuhl T.L. (2016). Distal Radius Hemiarthroplasty. J. Wrist Surg..

[43] Stegelmann S.D., Porter S., Yim J., Druessel L., Koepplinger M., Lee A. (2025). Outcomes of radial-sided wrist hemiarthroplasty: A systematic review. J. Orthop..

